# Heparin and Protamine Titration Does Not Improve Haemostasis after Cardiac Surgery: A Prospective Randomized Study

**DOI:** 10.1371/journal.pone.0130271

**Published:** 2015-07-02

**Authors:** Vladimir Radulovic, Anna Laffin, Kenny M. Hansson, Erika Backlund, Fariba Baghaei, Anders Jeppsson

**Affiliations:** 1 Department of Medicine/Hematology and Coagulation Disorders, Sahlgrenska University Hospital, Gothenburg, Sweden; 2 Department of Cardiothoracic Surgery, Sahlgrenska University Hospital, Gothenburg, Sweden; 3 AstraZeneca R&D, Mölndal, Sweden; 4 Department of Molecular and Clinical Medicine, Institute of Medicine, Sahlgrenska Academy, University of Gothenburg, Gothenburg, Sweden; Maastricht University Medical Center, NETHERLANDS

## Abstract

**Background:**

Bleeding complications are common in cardiac surgery. Perioperative handling of heparin and protamine may influence the haemostasis. We hypothesized that heparin and protamine dosing based on individual titration curves would improve haemostasis in comparison to standard dosing.

**Subjects and Methods:**

Sixty patients scheduled for first time elective coronary artery bypass grafting or valve surgery were included in a prospective randomized study. The patients were randomized to heparin and protamine dosing with Hepcon HMS Plus device or to standard weight and activated clotting time (ACT) based dosing. Blood samples were collected before and 10 minutes, 2 hours and 4 hours after cardiopulmonary bypass. Primary endpoint was endogenous thrombin potential in plasma 2 hours after surgery as assessed by calibrated automated thrombography. Secondary endpoints included total heparin and protamine doses, whole blood clot formation (thromboelastometry) and post-operative bleeding volume and transfusions. Heparin effect was assessed by measuring anti-Xa activity.

**Results:**

Endogenous thrombin potential and clot formation deteriorated in both groups after surgery without statistically significant intergroup difference. There were no significant differences between the groups in total heparin and protamine doses, heparin effect, or postoperative bleeding and transfusions at any time point. Significant inverse correlations between anti-Xa activity and endogenous thrombin potential were observed 10 min (r = -0.43, p = 0.001), 2 hours (r = -0.66, p<0.001) and 4 hours after surgery (r = -0.58, p<0.001).

**Conclusion:**

In conclusion, the results suggest that perioperative heparin and protamine dosing based on individual titration curves does not improve haemostasis after cardiac surgery. Postoperative thrombin generation capacity correlates to residual heparin effect.

**Trial Registration:**

www.isrctn.com ISRCTN14201041.

## Introduction

Cardiac surgery with cardiopulmonary bypass (CPB) imposes major trauma to the blood and activates the coagulation [1]. In order to prevent building of thrombus in the bypass circuit and minimize activation of the coagulation system, systemic heparinization has been used for decades. A common practice during CPB is administering a fixed, weight-based heparin dose and measuring the activated clotting time (ACT), targeting a certain level considered sufficiently safe for surgery. The ACT bioassay is influenced by many factors and the optimal level required is still unknown [2]. In order to improve heparin dosing regimen during CPB, a concept of estimating the individual heparin dose response has been developed [3], but the clinical results remain conflicting [4].

The impaired postoperative haemostasis in cardiac surgery patients is multifactorial [5]. Both reduced thrombin generation, impaired clot formation and reduced platelet function may contribute [6–8]. In standard cardiac surgery settings, we [9] and others [10–12] have found markedly decreased plasma potential to generate thrombin shortly after surgery, as determined by the calibrated automated thrombography (CAT) assay [13]. The reduced thrombin generation potential after surgery has been linked to increased postoperative bleeding [6,14]. Low thrombin generation potential after surgery has previously been associated with residual heparin effect [9,15].

Based on these findings we hypothesized that a more precise anticoagulation management during CPB, based on heparin and protamine titration, would be superior to weight based heparin dosing in terms of preservation of the haemostatic capacity. To test this hypothesis a prospective randomized study was designed.

## Material and Methods

### Ethics statement

The study was approved by the Regional Research Ethics Committee at Gothenburg university (Registry number 308–11) on April 18^th^, 2011. The study was registered before participant recruitment at the Regional Research Ethics Committee and after recruitment was started in The International Standard Randomized Controlled Trial Number (ISRCTN) registry (number ISRCTN14201041). Registration before start of recruitment was unfortunately overseen by the investigators.

### Patients

**Fig 1:**
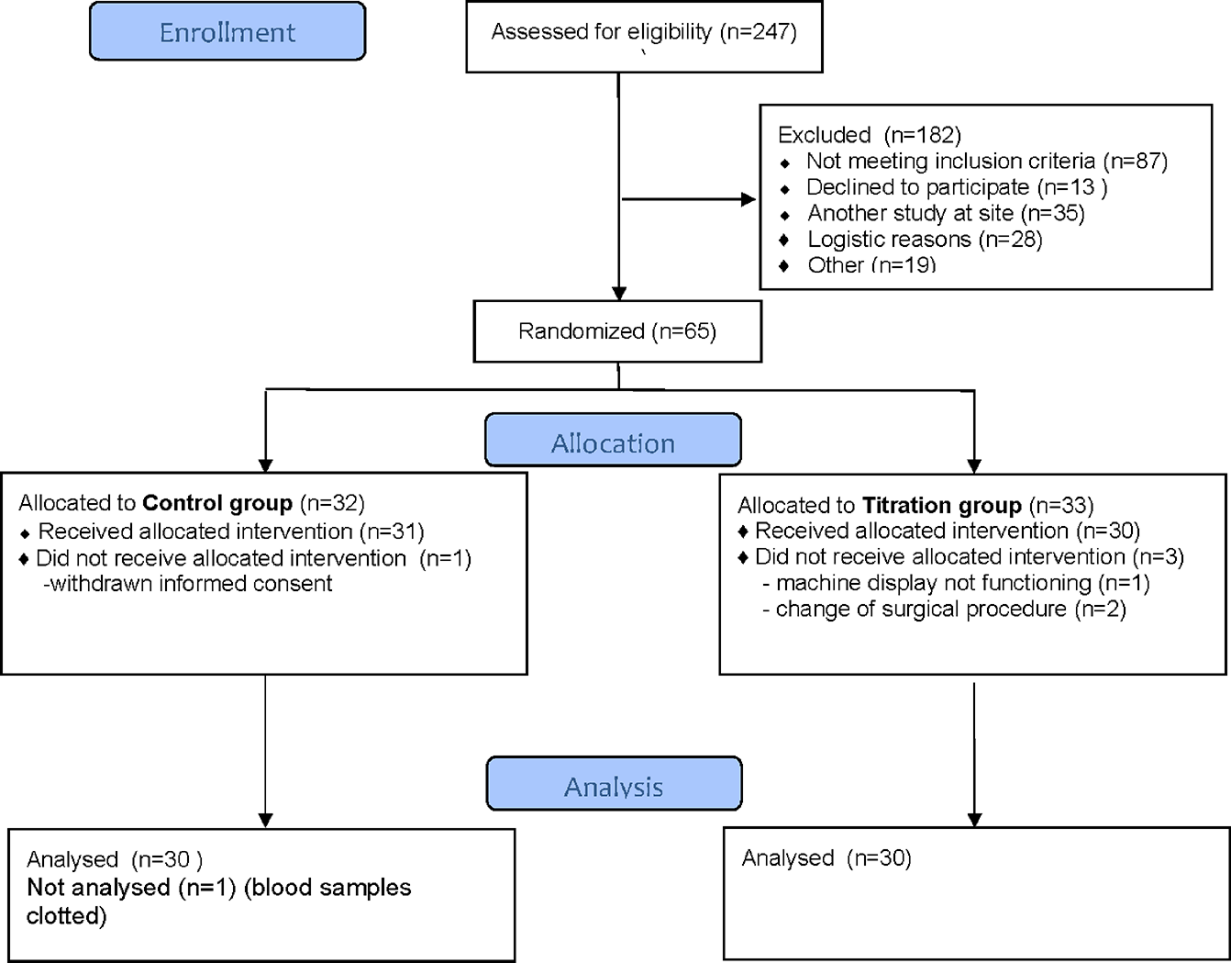
Flow chart over the study patients.

Initially two hundred thirty four adult patients (age > 18 years) were assessed for eligibility. One hundred seventy four did not meet the inclusion criteria or were excluded of other reasons (Fig 1). Predefined exclusion criteria were acute operation, previous cardiac surgery, known bleeding disorder, liver-, kidney- or psychiatric disease, previous stroke and/or thrombosis, treatment with a P2Y_12_ receptor antagonist >5 days before surgery and not being able to understand Swedish. Finally, sixty elective, first time coronary artery bypass grafting (CABG) or valve replacement surgery patients were included in the trial. All study participants provided written informed consent. Patient characteristics are given in Table 1.

**Table 1:** Patients’ characteristics at baseline.

	Titration group (n = 30)	Control group (n = 30)	p
Age (years)	66 ± 10	69 ± 8	0.19
Male	27 (90%)	28 (93%)	0.65
Weight (kg)	83 ± 12	82 ± 16	0.64
BMI (kg/m2)	27 ± 3	27 ± 4	0.83
EF (%)	57 ± 9	55 ± 10	0.61
Euroscore	3 (0–7)	4 (0–7)	0.12
Haemoglobin (g/L)	128 ± 12	128 ± 10	0.82
Platelet count (x10^9^/L)	221(144–366)	207(133–328)	0.62
aPTT (s)	35 (29–43)	36 (31–43)	0.28
PT INR	1.2 (1.0–1.2)	1.2 (1.0–1.3)	0.57
Haematocrit (%)	0.38 ± 0.04	0.38 ± 0.03	0.99
Antithrombin (kIU/L)	0.88 (0.73–1.19)	0.87 (0.66–1.15)	0.78
Aspirin (n)	28 (93%)	25 (83%)	0.24

Key: Data presented as mean ± standard deviation, median and range or number and percent. BMI–body mass index, EF–ejection fraction, aPTT–activated partial thromboplastin time, PT INR–prothrombin time INR. Wilcoxon-Mann-Whitney rank sum test or Student´s t-test.

### Study design

The study was a prospective, single center, randomized controlled trial. The investigation took place at Sahlgrenska University Hospital in Gothenburg, Sweden. Patients were recruited between October 25, 2011 and January 15, 2013. The study participants were randomized 1:1 (using opaque envelopes) immediately before surgery to either heparin and protamine titration using the heparin dose response curve (titration group) or weight based heparin and protamine dosing treatment, guided by activated clotting time (ACT) (control group). The perfusionist assigned to the case opened the envelope. Surgeons and intensive care unit (ICU) personnel were blinded to the randomization. The random allocation sequence was generated by a research assistant not otherwise involved in the study. This research assistant did not participate in registration of data or data analysis. The patients were enrolled by one of the co-authors (VR). Primary endpoint was endogenous thrombin potential in plasma 2 hours after surgery as assessed by calibrated automated thrombogram. Secondary endpoints included total heparin and protamine doses, whole blood clot formation (thromboelastometry), and post-operative bleeding volume and transfusions. The report of the study follows the CONSORT guidelines (S1 CONSORT Checklist). Study protocols are attached (S1 and S2 Protocols).

### Clinical management

Anaesthesia in all patients was induced with fentanyl and propofol followed by rocuronium and sevoflurane. The CPB circuit included a membrane oxygenator and roller pumps. Standard non-pulsatile CPB technique with moderate hypothermia (bladder temperature 34–36°C), and haemodilution was used. The CPB circuit was primed with 1400 ml of Ringer-Acetate (Fresenius Kabi AB, Uppsala, Sweden), and 200 ml of Mannitol (150 mg/ml)(Fresenius Kabi AB). Cardioprotection was achieved with intermittent antegrade cold blood cardioplegia. Weaning off CPB was performed after rewarming to a bladder temperature of 36°C. Heparin (10 000 IU) was added to the priming solution. Aspirin was not discontinued before surgery. All patients on aspirin received 75 mg once daily. None of the patients were treated with low molecular weight heparin or warfarin. Clopidogrel was discontinued at least five days before surgery. All patients received 2g tranexamic acid intravenously at anaesthesia induction and at the end of surgery. Aprotinin was not used in any of the study patients.

Blood products were transfused according to predefined transfusion protocol. Red blood cells were transfused if haemoglobin <70 g/L, haematocrit <20% or on-going bleeding. In bleeding patients red blood cells were transfused if the haemoglobin level was <100 g/L. Plasma was transfused in patients with ongoing bleeding (>200 ml/h) and coagulopathy documented by thromboelastography. Platelet concentrate was administered if on-going bleeding (>200 ml/h) and thrombocytopenia (<75 × 10^9^/L) or platelet dysfunction (e.g treatment with platelet inhibitor). The final decision regarding transfusions was left to the discretion of the responsible physician.

### Heparin management

In the titration group, HEPCON Haemostasis Management System Plus device (Medtronic Inc, Minneapolis, Minnesota) was used, according to manufacturer’s recommendations. After estimating the patients’ blood volume and individualized heparin sensitivity, the initial bolus heparin dose, heparin dose response and ACT were determined using a six channel cartridge (two channels with heparin concentration 2.5 U/ml, two with heparin concentration 1.5 U/ml and two without added heparin). Since the CPB circuit has already been primed with 10000 IU heparin, only bolus doses of heparin were estimated every 20 to 30 min throughout the surgery, in order to maintain target ACT above 480 s. At the end of the CPB, the protamine dose required for heparin neutralization was also established using the device and the effect controlled by an additional ACT check 10 min after weaning.

In the control group, the patients received unfractionated heparin (350 units/kg body weight) in order to achieve target activated clotting time (ACT) of more than 480 seconds. Heparin monitoring intraoperatively was performed by standard ACT (HEMOCHRON Jr. ACT+ [ITC, Edison, NJ]). After CPB, the heparin was reversed by administration of protamine sulfate (1 mg protamine/100 units of the initial heparin dose).

### Study procedures

The following pre- and perioperative variables were registered: age, gender, weight, body mass index (BMI), Euroscore, systolic ejection fraction, preoperative medication, number of grafts, CPB time, aortic clamp time, haemoglobin, haematocrit, platelet count, standard coagulation screening tests, ACT, antithrombin and anti-Xa plasma levels. Intraoperative blood loss was calculated by the operation nurse based on waste suction volume and number of used sponges. Postoperative bleeding was assessed as total amount of chest tube drainage during the first 12 postoperative hours.

Blood samples were collected at four time points: preoperatively, 10 minutes after weaning from CPB and two and four hours after surgery. The samples were drawn from a preoperatively placed central venous line after discarding the first 10 ml of blood. Specimens were collected in sodium citrate tubes (0.13M, 9 parts blood, 1 part sodium citrate), and centrifuged at 2000g for 20 min. The supernatant was filled in separate tubes and frozen at -70°C until further analysis. The samples for thrombin generation capacity studies were additionally centrifuged at 13000g for 10 min in order to obtain platelet poor plasma (PPP).

### Analyses

#### General

Heparin activity was assessed with anti-Xa and by the INTEM-CT:HEPTEM-CT ratio with thromboelastometry [16]. Coagulation was assessed with thrombin time (TT), ACT, aPTT, prothrombin time (PT INR) and thromboelastometry. All analyses, except HEPCON, activated clotting time (ACT) and rotational thromboelastometry were analyzed at the coagulation laboratory at Sahlgrenska University Hospital. The laboratory participates in the ECAT foundation external quality assessment programme (www.ecat.nl). Haemoglobin concentration, haematocrit and platelet count were analyzed with clinical standard methods.

#### Thrombin generation capacity

The Calibrated Automated Thrombography (CAT) assay was performed in round-bottom 96-well plates (Greiner microlon, U-shaped, high binding, USA). Citrated plasma samples (80 μl) and trigger solution (20 μl) (PPP reagent Cat# TS30.00, Thrombinoscope, Maastricht, The Netherlands) containing 5 pM tissue factor were mixed in sample wells. In parallel, a calibrator, (Cat # TS20.00, Thrombinoscope), was analyzed by mixing 20 μl of calibrator and 80 μl of pooled citrated normal human plasma in wells coupled to the sample wells. In addition, two controls were used on each plate, one with normal pooled plasma and the other with warfarin treated plasma with PT INR 2.0. Then the plate was moved to a fluorometer (Ascent reader, Thermolabsystems OY, Helsinki, Finland) and 20 μl of FluCa solution (Cat # TS50.00, Thrombinoscope) containing fluorogenic substrate and CaCl_2_ was dispensed by the instrument. The fluorogenic signal was measured at λex 390 nm, λem 460 nm during 60 min. The 96-well plate was kept on a 37°C heating block during additions of the reagents.

Start of thrombin activity (lag time), time to peak thrombin activity (TTP), peak thrombin activity (peak), and total endogenous thrombin potential (ETP) were calculated using the software Thrombinoscope (version 3.0.0.29) from Thrombinoscope BV (Maastricht, Netherlands).

#### Rotational thromboelastometry

Rotational thromboelastometry was performed on ROTEM delta instrument (TEM International, Munich, Germany) using 300 μl citrated whole blood, with previously described technique [17]. Assays using contact activation (INTEM), contact activation with addition of heparinase in order to neutralize the heparin effect (HEPTEM), tissue factor activation (EXTEM) and tissue factor activation with platelet inhibition to assess the fibrinogen status (FIBTEM) were run on all samples. Clotting time (CT) and maximum clot firmness (MCF) were evaluated with INTEM, EXTEM, HEPTEM and FIBTEM assays.

#### Coagulation assays

Anti-Xa activity was assessed by quantifying residual factor Xa activity by cleavage of a chromogenic substrate (STA-Liquid Anti Xa, Diagnostica Stago, Asnieres, France), reference value <0.05 kIU/L. Activated partial thromboplastin time (aPTT) was measured with STA APTT reagent containing cephalin as a source of the phospholipids and silica as a particulate activator (reference range 30–42 seconds). Determination of thrombin time (TT) was done by standard commercial STA Thrombin 2 test (Diagnostica Stago, Asnieres, France), adding thrombin to patient plasma and measuring the clotting time (reference range 14–21 seconds). Antithrombin was measured by chromogenic substrate method (STA-STACHROM AT III, Diagnostica Stago, Asnieres, France) (reference range 0.8–1.2 kIU/L). All analyses were performed on STA-R coagulometer (Diagnostica Stago, Asnieres, France).

### Statistics

A power analysis was done based on our own thrombin generation capacity data 2h after surgery [5]. The analysis showed that 25 patients in each group were needed to show a 30% difference in endogenous thrombin potential (ETP) between the groups 2h after surgery with 80% power and a significance level of 0.05. Results are expressed as mean and standard deviation (SD), median and range, or number and percent (%). Normality of distribution was tested by Shapiro-Wilk’s test. Student-T-test, Mann-Whitney U–test or Chi-square test was used to compare groups. Statistical significance was generally defined as a p value of <0.05. Correlation between anti-Xa levels, CAT variables and INTEM-CT:HEPTEM-CT was analyzed by Spearman’s rank correlation test.

Differences between control and titration group, with respect to CAT, ROTEM, antithrombin, anti-Xa, ACT, haemoglobin, platelet count, PT INR, aPTT and TT analyzed at different time points, were compared using analysis of variance (ANOVA) for repeated measurements independently of data distribution. Student-T-test was used to test the different time points if the ANOVA analysis indicated a significant difference between groups (p<0.05). STATISTICA 10 software was used for statistical analyses (StatSoft, Tulsa, OK, USA).

## Results

### General

All the study participants completed the study and there was no missing data. There was no difference between titration control and group in any of the baseline variables, Table 1. Despite randomization, aortic clamp time and ECC time were significantly longer in the control group than in the titration group, Table 2. Three patients (5%) were re-explored because of bleeding (one in the titration group and two in the control group). Two patients (both in control group) were reoperated because of early myocardial ischemia. There were no other thromboembolic complications and all patients could be discharged from the hospital with no further complications.

**Table 2:** Intra- and postoperative patients’ characteristics in the titration group and the control group.

	Titration group(n = 30)	Control group(n = 30)	p
Extra fluid during ECC (ml)	623 ± 568	680 ± 559	0.70
Anastomoses	3 (0–4)	3 (0–6)	0.11
Valve surgery (n)	2 (7%)	5 (17%)	0.23
Initial ACT (s)	125 ± 16	117 ± 12	0.03*
Initial heparin bolus (IU)	35000 (20000–55000)	30000(20000–40000)	0.009**
ACT after initial heparin bolus (s)	660 ± 30	552 ± 10	0.01*
Extra heparin during CPB (IU)	0 (0–10000)	2500 (0–35000)	0.10
Total heparin dose (IU)	37150 ± 8734	37167 ± 11573	0.99
Protamine/heparin ratio	0.86 ± 0.14	0.84 ± 0.19	0.57
Total protamine dose (mg)	319 ± 96	314 ± 58	0.78
Clamp time (min)	44 ± 3	57 ± 4	0.009**
ECC time (min).	69 ± 4	87 ± 6	0.023*
Intraoperative bleeding (ml)	325 (100–850)	300 (100–1200)	0.90
Bleeding at 12 hours (ml)	475(300–1070)	495(150–1460)	0.81
RBC transfusions (units)	0(0–6)	0(0–6)	0.08
FFP transfusions (units)	0(0–3)	0(0–5)	0.56
PLT transfusions (units)	0(0–2)	0(0–3)	0.63
ICU stay (hours)	6.9 (4–21.3)	5.9 (5.1–21.5)	0.25
Ventilator support (hours)	3 (2–4)	3 (2–4)	0.23

Key: Data presented as mean and standard deviation, median and range or number and percent. ECC–extracorporeal circulation, ACT–activated clotting time, RBC–red blood cells, FFP–fresh frozen plasma, PLT–platelets, ICU–intensive care unit. Wilcoxon-Man-Whitney rank sum test or Student´s t-test.

* p<0.05

** p<0.01

### Heparin and protamine doses

The titration group received higher bolus dose heparin (p = 0.009), but the total heparin doses did not differ between the groups (p = 0.99). Patients in the titration group had higher mean ACT after heparin bolus dose (p = 0.01), Table 2. Fourteen patients in control- and twelve patients in titration group received extra heparin during surgery. One patient in each group did not reach ACT 480 s after the initial heparin bolus dose. The total protamine dose did not differ significantly (p = 0.78).

### Thrombin generation potential

There was a significant reduction in thrombin generation capacity variables (lag time, time to peak, peak value and ETP) in both groups postoperatively compared to the preoperative measurements, Table 3. The lowest ETP was registered 4 hours after CPB in both groups. Time to peak (TTP) was significantly longer in the titration group 4 hours after surgery but no other statistically significant difference between the groups was observed.

**Table 3:** Repeated measures analysis of variance (ANOVA) for thrombin generation capacity variables in the titration group and in the control group preoperatively and 10min, 2h and 4h after cardiopulmonary bypass.

	Titration group	Control group	p value group	p value time	p value group/time interaction
**Lag time (min)**			0.20	< 0.001	0.95
Preoperative	3.0 (2.0–4.0)	2.7 (1.7–3.3)			
10 min after CPB	5.0 (3.0–14.0)	5.0 (2.0–8.0)			
2 hours after CPB	5.2 (3.2–8.5)	4.7 (2.4–6.7)			
4 hours after CPB	4.7 (3.0–9.0)	3.8 (3.0–7.0)			
**Time to peak (min)**			0.016	< 0.001	0.17
Preoperative	6.7 (5–9)	6.3 (4–7)			
10 min after CPB	9.8 (5.7–19.3)	9.9 (5.0–12.2)			
2 hours after CPB	10.5 (7–14.7)	8.6 (5.0–11.7)			
4 hours after CPB	13.1 (7.8–18.3)	10.0 (5.9–13.5)			
**Peak (nM)**			0.4	< 0.001	0.52
Preoperative	194 (123–286)	195 (125–257)			
10 min after CPB	70 (0–202)	70 (0–216)			
2 hours after CPB	14 (0–129)	43 (0–156)			
4 hours after CPB	1 (0–152)	0 (0–155)			
ETP (nM*min)			0.38	< 0.001	0.41
Preoperative	1322 (915–1813)	1305 (855–2047)			
10 min after CPB	635 (0–1328)	631 (0–1108)			
2 hours after CPB	183 (0–1099)	440 (0–1125)			
4 hours after CPB	12 (0–1065)	0 (0–1096)			

Key: Data presented as mean and standard deviation or median and range. ETP–endogenous thrombin potential, CPB–cardiopulmonary bypass.

### Coagulation

All coagulation tests except PT INR and ACT indicated a moderately impaired coagulation after surgery without any significant intergroup differences, Tables 4 and 5. Clotting time measured with thromboelastometry, aPTT and thrombin time were prolonged postoperatively while antithrombin levels were lower in comparison to the preoperative measurements.

**Table 4:** Repeated measures analysis of variance (ANOVA) for haemostatic variables in the titration group and in the control group preoperatively and 10min, 2h and 4h after cardiopulmonary bypass.

	Titration group	Control group	p value group	p value time	p value group/time interaction
**Antithrombin (kIU/L)**			0.78	< 0.001	0.36
Preoperative	0.88 (0.73–1.19)	0.86 (0.66–1.15)			
10 min after CPB	0.69 (0.37–0.89)	0.69 (0.53–0.93)			
2 hours after CPB	0.73 (0.60–1.00)	0.73 (0.50–1.10)			
4 hours after CPB	0.76 (0.60–0.90)	0.76 (0.48–1.04)			
**Anti Xa (kIU/L)**			0.29	< 0.001	0.87
Preoperative	0.04 ± 0.09	0.05 ± 0.09			
10 min after CPB	0.05 ± 0.11	0.09 ± 0.11			
2 hours after CPB	0.09 ± 0.11	0.13 ± 0.15			
4 hours after CPB	0.14 ± 0.13	0.16 ± 0.15			
**ACT (s)**			0.99	0.41	0.036
Preoperative	125 ± 16*	117 ± 12			
10 min after CPB	116 ± 14*	123 ± 12			
2 hours after CPB	122 ± 10	126 ± 12			
4 hours after CPB	121 ± 12	124 ± 12			
**Haemoglobin (g/L)**			0.7	<0.001	0.52
Preoperative	128 ± 12	128 ± 10			
10 min after CPB	104 ± 11	107 ± 10			
2 hours after CPB	109 ± 16	107 ± 18			
4 hours after CPB	109 ± 14	111 ± 13			
**Platelet count (x109/L**					
Preoperative	227 ± 59	217 ± 4	0.63	<0.001	0.96
10 min after CPB	152 ± 44	148 ± 57			
2 hours after CPB	188 ± 65	183 ± 49			
4 hours after CPB	196 ± 60	185 ± 46			
**PT (INR**					
Preoperative	1.2 ± 0.1	1.2 ± 0.1	0.47	<0.001	0.98
10 min after CPB	1.3 ± 0.1	1.3 ± 0.1			
2 hours after CPB	1.3 ± 0.1	1.3 ± 0.1			
4 hours after CPB	1.2 ± 0.1	1.3 ± 0.1			
**aPTT (s)**					
Preoperative	36 ± 3	36 ± 3	0.91	<0.001	0.94
10 min after CPB	39 ± 6	39 ± 4			
2 hours after CPB	41 ± 7	40 ± 7			
4 hours after CPB	46 ± 11	46 ± 11			
**Thrombin time (s)**			0.59	<0.001	0.79
Preoperative	17 ± 1	17 ± 1			
10 min after CPB	48 ± 108	39 ± 62			
2 hours after CPB	33 ± 31	34 ± 48			
4 hours after CPB	64 ± 111	47 ± 47			

Key: Data presented as mean and standard deviation or median and range. ACT–activated clotting time, aPTT–activated partial thromboplastin time CPB–cardiopulmonary bypass, INR–International normalized ratio, PT–prothrombin time.

**Table 5:** Repeated measures analysis of variance (ANOVA) for thromboelastometric variables in the titration group and in the control group preoperatively and 10min, 2h and 4h after cardiopulmonary bypass.

	Titration group	Control group	p value group	p value time	p value group/time interaction
**INTEM CT (s)**			0.13	< 0.001	0.051
Preoperative	158 (113–237)	159 (116–244)			
10 min after CPB	183 (136–274)	191 (145–235)			
2 hours after CPB	190 (136–290)	177 (136–248)			
4 hours after CPB	192 (147–406)	180 (160–229)			
**EXTEM CT (s)**			0.33	< 0.001	0.43
Preoperative	47 (28–59)	46 (35–197)			
10 min after CPB	59 (44–95)	66 (52–102)			
2 hours after CPB	53 (43–64)	52 (43–70)			
4 hours after CPB	56 (37–74)	50 (34–128)			
**INTEM-MCF (mm)**			0.25	<0.001	0.66
Preoperative	65 (57–78)	64 (51–75)			
10 min after CPB	59 (41–76)	58 (46–67)			
2 hours after CPB	61 (51–78)	60 (50–70)			
4 hours after CPB	62 (55–81)	61 (54–71)			
**EXTEM MCF (mm)**			0.36	<0.001	0.92
Preoperative	65 (57–78)	64 (51–75)			
10 min after CPB	59 (41–76)	58 (46–67)			
2 hours after CPB	61 (51–78)	60 (50–70)			
4 hours after CPB	62 (55–81	61 (54–71)			
**HEPTEM MCF (mm)**			0.16	<0.001	0.64
Preoperative	62 (55–76)	61 (51–71)			
10 min after CPB	57 (40–72)	56 (48–67)			
2 hours after CPB	59 (50–76)	58 (50–67)			
4 hours after CPB	61 (50–76)	59 (53–70)			
**FIBTEM MCF (mm)**			0.34	<0.001	0.55
Preoperative	17 (9–36)	16 (9–25)			
10 min after CPB	14 (4–34)	12 (6–21)			
2 hours after CPB	14 (6–40)	11 (5–20)			
4 hours after CPB	15 (6–55	12 (4–21)			

Key: Data presented as mean and standard deviation or median and range. CPB–cardiopulmonary bypass.

### Postoperative heparin effect

Anti-Xa activity was undetectable before surgery but increased postoperatively in both groups without significant intergroup difference, Table 4. The highest activity was registered 4 hours after CPB. Similarly, the INTEM/HEPTEM ratio increased postoperatively in both groups without significant intergroup difference, Table 5.

### Correlation between thrombin generation capacity and heparin effect

There were significant inverse correlations between anti-Xa levels and ETP 10 min (r = -0.43, p = 0.001), 2 hours (r = -0.66, p<0.001) and 4 hours after surgery (r = -0.58, p<0.001). Postoperative INTEM-CT:HEPTEM-CT ratio correlated also with ETP levels two hours and (r = 0.44; p = 0.001) and four hours (r = 0.44, p = 0.001) after surgery.

### Bleeding and transfusions

There were no significant differences in intraoperative and postoperative bleeding or in transfusion rates between the two groups, Table 2. The groups did not differ in haemoglobin level at any time point, Table 3.

## Discussion

In the present randomized study we hypothesized that heparin and protamine dosing based on individual titration curves would improve postoperative haemostasis. This hypothesis was grounded on the previously demonstrated association between thrombin generation capacity and residual heparin effect and on data indicating that titration optimizes heparin and protamine dosing. However, the results did not support our hypothesis since both thrombin generation capacity and all other haemostatic tests were comparable in the two groups.

There have been a few previous studies with similar design addressing the effect of heparin titration on thrombin generation. Hofmann et al [18] found decreased thrombin-antithrombin complex (TAT) levels with the Hepcon device and Koster et al [19] found lower TAT, prothrombin fragment 1.2 and D-dimer levels. TAT and prothrombin fragment 1.2 reflect the amount of thrombin that already has been produced and not the ability of plasma to further generate thrombin. The latter is assessed by calibrated automated thrombography, a method used in the present study. Therefore our study is not directly comparable to previous trials using other markers of thrombin generation. Furthermore, the mode of action of heparins in clinical settings is far from clear, still generating a debate whether it is increased thrombin inactivation or decreased prothrombin consumption that is a primary effect [20,21]. While low heparin concentrations facilitate thrombin binding to antithrombin at expense of its binding to other plasma proteins (resulting in increased TAT), it has been suggested that heparin´s primary effect in the setting of higher heparin concentrations is inhibition of prothrombin consumption [22]. The latter scenario is probably more relevant for cardiac surgery. That implies that measuring TAT may be unsuitable to study the action of heparin in cardiac surgery.

A number of studies have addressed the issue of individualized heparin and protamine titration with other endpoints than thrombin generation capacity. The results have been conflicting [4]. A recent meta-analysis of four randomized controlled trials demonstrated reduced postoperative blood loss with heparin and protamine titration compared to standard dosing [23]. This could however not be confirmed in the present study, where postoperative bleeding did not differ between the two groups. It should be noted that present study lacks statistical power in clinical endpoints and the results should therefore be interpreted very cautiously. Nonetheless, the absence of effect on postoperative bleeding is in accordance with two other recently published trials [18,24].

With the present results, we could confirm previous findings of incomplete heparin reversal and/or heparin rebound during the first couple of hours after surgery [9]. The residual heparin effects after surgery could also be shown using the ratio between INTEM-CT:HEPTEM-CT, as described by Mittermayr et al [16]. In a recent report, residual plasma heparin was detected in 88% of all postoperative samples [25]. Also in a present study we found a significant correlation between anti-Xa levels and ETP, indicating that residual heparin activity contributes to the marked reduction in thrombin generation capacity during the early postoperative period after cardiac surgery.

Some previous studies have described higher total heparin and lower total protamine dosage using heparin and protamine titration compared to weight and ACT-based dosing [26,27]. In the present study no difference was observed. Although the initial heparin bolus dose given was higher in the titration group than in the control group, the control group received more heparin intra-operatively resulting in comparable total doses. The lack of effect on total protamine doses could at least partly be explained by the fact that in the present study heparin levels were not allowed to drop towards the end of CPB which otherwise is common. The final ratio between total protamine and heparin dose was 0.86 in this study. This is higher than in a comparable study [28] but still lower than the traditionally used protamine:heparin ratios (1:1 or 1.3:1). Taneja et al have recently demonstrated in vitro that anti-IIa activity of heparin is more resistant to neutralization by protamine in comparison to anti-Xa activity and that higher protamine doses are thus needed [29].

There are several limitations of the study. All personnel could not be completely blinded to randomization. The sample tubes did not contain corn trypsin inhibitor (CTI) in order to block sample activation by the contact pathway. However, this should not be necessary, giving the concentration of the tissue factor (TF) used (5 pM) [30]. The TF concentration in itself is also debatable. The concentration we utilized has been used in several studies [10,12,31] and our sampling took place after heparin reversal. In spite of randomization, the control group had somewhat longer CPB and clamp time. However, this did not affect thrombin generation capacity, coagulation or bleeding despite of exposure to longer surgical trauma. Furthermore, the study lacks statistical power in clinical endpoints. Finally, this is a single center study and whether the results can be generalized to other centers remain elusive.

In conclusion, protamine and heparin titration did not improve postoperative haemostasis compared to routine practice of weight based heparin dosing. No beneficial effects on postoperative bleeding or transfusions requirements were observed. Larger randomized studies comparing clinically important outcome variables between heparin titration and standard dosing are warranted.

## Supporting Information

S1 CONSORT Checklist CONSORT checklist. (DOC)

S1 Protocol Trial protocol (Swedish). (DOC)

S2 Protocol Trial protocol (English summary). (DOC)
