# Supplementary material for: Heparin and Protamine Titration Does Not Improve Haemostasis after Cardiac Surgery: A Prospective Randomized Study
Source: PLoS One. 2015 Jul 2;10(7):e0130271. doi: 10.1371/journal.pone.0130271 (PMC4489911; doi:10.1371/journal.pone.0130271)
Supplement: S1 Protocol — (DOC) [file pone.0130271.s002.doc]

ACT-STYRD HEPARINDOSERING ELLER HEPARINTITRERING: EFFEKTER PÅ ENDOGEN TROMBINGENERERINGSKAPACITET EFTER HJÄRTKIRURGI

FORSKNINGSPLAN

**SAMMANFATTNING**

Blödningskomplikationer är vanliga under och efter hjärtkirurgi. En faktor som skulle kunna leda till blödning är en bristande förmåga att bilda trombin då trombins spjälkning av fibrinogen är en nyckelprocess i koagulationskaskaden. Den endogena kapaciteten att generera trombin är påtagligt reducerad i det tidiga postoperativa skedet efter hjärtkirurgi. Egna data tyder på att en kvarstående heparineffekt kan bidra till reduktionen i trombinbildning. Heparin används under hjärtoperationer för att inte det skall bildas koagler i hjärt-lungmaskinen. Den aktuella studiens syfte är att jämföra två olika system för heparindosering på kvarvarande heparineffekt och förmåga att generera trombin efter hjärtkirurgi. Sextio patienter inkluderas i en prospektiv randomiserad studie där hälften av patienterna randomiseras till heparindosering efter activated clotting time (ACT) och hälften till dosering efter heparintitrering. Plasmaprover analyseras före och omedelbart efter operation samt två och fyra timmar senare. Primär endpoint är endogen trombingenereringskapacitet 2h efter avslutad operation.

**BAKGRUND**

Årligen hjärtopereras 6000 vuxna och 500 barn i Sverige (1). Resultaten är generellt sett utmärkta men en del patienter drabbas dessvärre av komplikationer under och efter operationen. En av de vanligaste komplikationerna är blödning. Trots framsteg inom kirurgisk teknik och ökad kunskap om faktorer som påverkar blodets koagulation blöder 5-7% av patienterna så mycket att de behöver reopereras efter operationen och 50-60% behöver blodtransfusioner under vårdtiden (1,2). Reoperation för blödning är en oberoende riskfaktor för död inom 30 dagar efter operationen och är associerad med en 2-3-faldigt ökad risk (2). Patientens blodförluster och därmed följande transfusioner innebär inte bara en ökad risk för postoperativ morbiditet och mortalitet utan är också förenat med höga kostnader för samhället för blod och koagulationsprodukter. Det finns således all anledning att försöka begränsa hjärtopererade patienters perioperativa blödning och transfusioner.

Blödningen under och efter hjärtkirurgi är multifaktoriell. Både kirurgiska faktorer och en försämrad hemostas kan bidra. Hemostasen påverkas av blodets passage genom hjärtlungmaskinen, hemodilution och hypotermi, men också av det kirurgiska traumat och pre- och perioperativ medicinering, exempelvis trombocythämmare och heparin (3-5).

En faktor som kan påverka risken för blödning efter hjärtkirurgi är patientens produktion eller förmåga att bilda trombin (6,7). Trombin är en nyckelfaktor i koagulationskaskaden som spjälkar fibrinogen till fibrin. Trombin har mycket kort halveringstid och är svårt att bestämma i blodprover varför de flesta analysmetoder bygger på att mäta mer stabila nedbrytningsprodukter, t ex trombin-antitrombinkomplex (TAT). Ett annat sätt är att mäta den endogena trombingenerationskapaciteten genom att tillsätta tissue factor som inducerar trombinbildning till ett plasmaprov och sedan direkt mäta trombinfrisättningen (8). I en nyligen avslutad studie fann vi att den endogena trombingenererings-kapaciteten är påtagligt reducerad i det tidiga postoperativa skedet efter hjärtkirurgi (9). Vi fann också att reduktion åtminstone delvis kunde förklaras av en kvarstående heparineffekt. Heparin är en potent koagulationshämmare som ges till patienter i höga doser under operationer med hjärtlungmaskin för att undvika koagelbildning i hjärtlungmaskinen. Heparindoseringen styrs vanligen genom att man följer activated clotting time (ACT) i blodet. Efter operationen reverseras heparineffekten med protamin. Det finns också nyare system (HEPCON, Hemochron) som mer detaljerat titrerar fram vilka heparin och protamindoser som är nödvändiga.

**HYPOTES**

Heparintitrering för att styra heparindoseringen leder till bättre bevarad trombingenereringskapacitet efter hjärtkirurgi.

**FRÅGESTÄLLNINGAR**

**Primär**

1. Förbättrar heparintitrering den endogena trombingenereringskapaciteten två timmar efter hjärtkirurgi jämfört med ACT-styrd heparindosering?

**Sekundära**

1. Vilket heparindoseringsprotokoll ger minst kvarstående heparineffekt efter kranskärlskirurgi?
2. Påverkar heparindoseringsprotokollet postoperativ blödning och transfusionsbehov eller doser av heparin och protamin?
3. Påverkar heparindoserinsgsprotokolet helblodskoagulation?

**STUDIEDESIGN**

Prospektiv öppen randomiserad och kontrollerad studie. Studien är prövarinitierade och finansieras med forskningsanslag.

**INKLUSIONS OCH EXKLUSIONSKRITERIER**

Patienter som skall genomgå förstagångs kranskärlsoperation eller operation av en hjärtklaff på Sahlgrenska Universitetssjukhuset, äldre än 18 år, inkluderas. Patienter som har känd blödningsrubbning, har känd lever- eller njursjukdom, tidigare stroke eller har pågående behandling med andra mediciner som påverkar blödning och koagulation än acetylsalicylsyra exkluderas. Clopidogrel (P2Y12 receptor antagonist) skall vara utsatt minst fem dagar före operation.

**GENOMFÖRANDE**

Hjärtsjuka patienter (n=60) som accepterats för operation inkluderas efter informerat samtycke. Hälften av patienterna randomiseras före operation till viktbaserad heparin och protamindosering efter ACT värden mätt med Hemochron Jr, ACT+ instrument, vilket är standardmetod på Sahlgrenska. För den andra hälften patienter doseras heparin och protamin efter bestämning av individuell heparinkänslighet och utifrån heparintitrering med HEPCON (Haemostasis management system device) instrument. Blodprover tas från en central venkateter före operation samt 10 minuter, 2h och 4h efter protaminisering. Totalt samlas 80 ml blod från varje patient. Följande analyser genomförs vid samtliga tillfällen: endogen trombingenereringskapacitet, ACT, anti-FXa, trombintid, antitrombin och tromboelastometri (med och utan heparinas). Alla perioperativa heparindoser, protamindoser, ACT värden samt postoperativ blödning och transfusioner registreras.

**STATISTISK PLAN**

Baserat på egna data har vi genomfört en powerberäkning som innebär att det krävs 25 patienter per grupp för att visa en 30% skillnad mellan grupperna i endogenous thrombin generation potential (ETP) med 80% power och en signifikansnivå på 0.05.

**PUBLIKATION**

Resultatet av studien kommer att presenteras i en internationell tidskrift med peer-review-system.

# REFERENSER

1. Swedeheart Årsrapport 2009. www.ucr.uu.se/swedeheart/index.php/arsrapporter
2. Dacey LJ, Munoz JJ, Baribeau YR, Johnson ER, Lahey SJ, Leavitt BJ, Quinn RD, Nugent WC, Birkmeyer JD, O'Connor GT. Reexploration for hemorrhage following coronary artery bypass grafting: incidence and risk factors. Northern New England Cardiovascular Disease Study Group. Arch Surg 1998;133:442-7.
3. Despotis GJ, Avidan MS, Hogue CW, Jr. Mechanisms and attenuation of hemostatic activation during extracorporeal circulation. Ann Thorac Surg 2001;72:S1821-31.
4. Hartmann M, Sucker C, Boehm O, Koch A, Loer S, Zacharowski K. Effects of cardiac surgery on hemostasis. Transf Med Rev 2006;20:230-41.
5. Paparella D, Brister SJ, Buchanan MR. Coagulation disorders of cardiopulmonary bypass: a review. Intensive Care Med 2004;30:1873-81.
6. Edmunds LH, Jr., Colman RW. Thrombin during cardiopulmonary bypass. Ann Thorac Surg 2006;82(6):2315-2322
7. Swords Jenny N, Mann KG. Thrombin. In: Colman RW, Hirsch J, Marder VJ, Clowes AW, George JN ed. Hemostasis and thrombosis, basic principles and clinical practice, 4th ed: Lippincott Williams and Wilkins;Philadelphia; 2001.
8. Hemker HC, Giesen P, AlDieri R, Regnault V, de Smed E, Wagenvoord R, Lecompte T, Beguin S. The calibrated automated thrombogram (CAT): a universal routine test for hyper- and hypocoagulability. Pathophysiol Haemost Thromb 2002; 32(5-6):249-253
9. Radulovic V, Hyllner M, Ternström L, Karlsson M, Bylock A, Hansson KM, Bagahei F, Jeppsson A. Heparin contributes to reduced plasma trombin generation capacity early after cardiac surgery. Submitted
