# Supplementary material for: Heparin and Protamine Titration Does Not Improve Haemostasis after Cardiac Surgery: A Prospective Randomized Study
Source: PLoS One. 2015 Jul 2;10(7):e0130271. doi: 10.1371/journal.pone.0130271 (PMC4489911; doi:10.1371/journal.pone.0130271)
Supplement: S2 Protocol — (DOC) [file pone.0130271.s003.doc]

ACT GUIDED HEPARIN DOSING OR HEPARIN TITRATION: EFFECTS ON ENDOGENOUS THROMBIN GENERATION CAPACITY AFTER CARDIAC SURGERY

STUDY PLAN

**SUMMARY**

Bleeding complications are common during and after cardiac surgery. One of the factors that could cause bleeding is decreased thrombin, since its cleavage of fibrinogen is one of the key steps in the coagulation process. Endogenous thrombin generation capacity is markedly reduced in the early postoperative stage. Our own data suggest that residual heparin effect could contribute to decreased thrombin potential. Heparin is used in cardiac surgery in order to prevent clot occurence in heart-lung machine. The purpose of this study is to compare two different ways of heparin dosing in regard to residual heparin effect and ability to generate thrombin after surgery. Sixty patients are included in a prospective randomized study where one half is randomized to heparin dosing using activated clotting time (ACT), while in the other half heparin is dosed by heparin titration. Plasma samples are analyzed before, immediately after surgery and two and four hours later. The primary endpoint is endogenous thrombin generation capacity two hours after operation.

**BACKGROUND**

Cardiac surgery is performed on six thousend adults and five hundred children in Sweden annually (1). Although the results are excellent in general, a minor part of the patients still suffer complications during and after surgery. The most common complication is bleeding. In spite of surgical development and better knowledge on haemostasis, 5-7% of patients are reoperated on due to excessive haemorrhage after surgery and 50-60% need blood transfusions during hospitalization (1,2). Reoperation due to haemorrhage is an independent risk factor for 30 days mortality, increasing that risk 2-3 fold (2). Besides the increased morbidity and mortality risk, the postoperative bleeding and transfusions pose also high costs for society. There is thus a need for trying to limit the postoperative bleeding and transfusions.

Bleeding during and after cardiac surgery is multifactorial. Both the surgical factors and deranged haemostasis contribute. The haemostatic balance is influenced by heart- lung machine, haemodilution and hypothermia but also surgical trauma and pre- and postoperative medication, for instance the platelet blockers and heparin (3-5).

One of the factors that could affect the bleeding risk is ability to synthesize thrombin (6,7). Thrombin is the key factor in the coagulation process and it cleaves fibrinogen to fibrin. Due to the short half life of thrombin, it is difficult to direct measure it. Instead, the more stabile thrombin degradation products, like thrombin-antithrombin complex (TAT) are determined. Another way of measuring the endogenous thrombin is adding the tissue factor to plasma sample and measuring its thrombin generation capacity (8). In a previous study our group found markedly reduced thrombin generation capacity in the early postoperative stage (9). That finding could be attributed to residual heparin effect. Heparin is a potent anticoagulant, added to heart-lung machine in order to prevents blood clots. Heparin dosing is usually guided by determining activated clotting time (ACT). Its effect is reversed by protamine after surgery. There is also a more complex technique using HEPCON instrument, which titrates the right dose of heparin and protamine.

**HYPOTHESIS**

Heparin dosing using heparin titration leads to better preserved thrombin generation capacity after cardiac surgery.

**Endpoints**

**Primary**

1. Does heparin titration increase the endogenous thrombin generation capacity at two hours after cardiac surgery compared to ACT-guided heparin dosing?

**Secondary**

1. Which heparin dosing protocol gives less residual heparin activity after cardiac surgery?
2. Does heparin dosing protocol affect the postoperative bleeding, transfusions or doses of heparin and protamine?
3. Does heparin dosing protocol affect the whole blood coagulation ability?

**STUDY DESIGN**

The prospective, open, randomized and controlled study. It is researcher initiated and research funds financed.

**INCLUSION AND EXCLUSION CRITERIA**

Patients undergoing first time coronary artery bypass grafting (CABG) or heart valve surgery at Sahlgrenska University Hospital, age > 18 years are included. Patients with known bleeding disorder, liver or kidney disease, previous stroke or on-going treatment with medications that can affect bleeding tendency are excluded. Clopidogrel (P2Y12 receptor antagonist) is to be stopped at least five days prior to surgery.

**REALISATION**

Patients with heart disease (n=60) accepted for surgery are included after obtaining written informed consent. One half of the patients is randomized before operation to weight based heparin and protamine dosing , guided by ACT determined by Hemochron Jr, ACT+ instrument, the standard method at our institution. The other half is dosed after detemining the individual heparin sensitivity and using heparin and protamine titration by HEPCON (Haemostasis management system device). Blood samples are taken from a previously inserted central venous line, before and 10 minutes , two hours and four hours after protamine. Totally 80 ml blood is drawn from each study patient. These tests are performed with all sampling occassions: endogenous thrombin generation capacity, ACT, anti-FXa, thrombin time, antithrombin and rotational thromboelastometry (with and without heparnase added). All peroperative heparin and protamine doses, ACT values, postoperative bleeding and transfusions are registered.

**STATISTICS**

Power calculation is based on our own data. It takes 25 patients in each group to show 30% difference between the groups in endogenous thombin generation potential (ETP), with 80% power and significance level 0.05.

**PUBLICATION**

The study results are going to be presented in an international journal with peer-review system.

# REFERENCES

1. Swedeheart Årsrapport 2009. www.ucr.uu.se/swedeheart/index.php/arsrapporter
2. Dacey LJ, Munoz JJ, Baribeau YR, Johnson ER, Lahey SJ, Leavitt BJ, Quinn RD, Nugent WC, Birkmeyer JD, O'Connor GT. Reexploration for hemorrhage following coronary artery bypass grafting: incidence and risk factors. Northern New England Cardiovascular Disease Study Group. Arch Surg 1998;133:442-7.
3. Despotis GJ, Avidan MS, Hogue CW, Jr. Mechanisms and attenuation of hemostatic activation during extracorporeal circulation. Ann Thorac Surg 2001;72:S1821-31.
4. Hartmann M, Sucker C, Boehm O, Koch A, Loer S, Zacharowski K. Effects of cardiac surgery on hemostasis. Transf Med Rev 2006;20:230-41.
5. Paparella D, Brister SJ, Buchanan MR. Coagulation disorders of cardiopulmonary bypass: a review. Intensive Care Med 2004;30:1873-81.
6. Edmunds LH, Jr., Colman RW. Thrombin during cardiopulmonary bypass. Ann Thorac Surg 2006;82(6):2315-2322
7. Swords Jenny N, Mann KG. Thrombin. In: Colman RW, Hirsch J, Marder VJ, Clowes AW, George JN ed. Hemostasis and thrombosis, basic principles and clinical practice, 4th ed: Lippincott Williams and Wilkins;Philadelphia; 2001.
8. Hemker HC, Giesen P, AlDieri R, Regnault V, de Smed E, Wagenvoord R, Lecompte T, Beguin S. The calibrated automated thrombogram (CAT): a universal routine test for hyper- and hypocoagulability. Pathophysiol Haemost Thromb 2002; 32(5-6):249-253
9. Radulovic V, Hyllner M, Ternström L, Karlsson M, Bylock A, Hansson KM, Bagahei F, Jeppsson A. Heparin contributes to reduced plasma trombin generation capacity early after cardiac surgery. Submitted.
